# Historical Parallels, Ebola Virus Disease and Cholera: Understanding Community Distrust and Social Violence with Epidemics

**DOI:** 10.1371/currents.outbreaks.aa1f2b60e8d43939b43fbd93e1a63a94

**Published:** 2016-01-26

**Authors:** Samuel Cohn, Ruth Kutalek

**Affiliations:** School of Humanities, University of Glasgow, and Honorary Fellow, IASH, Edinburgh, Scotland

**Keywords:** cholera, community engagement, ebola, resistance, violence

## Abstract

In the three West African countries most affected by the recent Ebola virus disease (EVD) outbreak, resistance to public health measures contributed to the startling speed and persistence of this epidemic in the region. But how do we explain this resistance, and how have people in these communities understood their actions? By comparing these recent events to historical precedents during Cholera outbreaks in Europe in the 19th century we show that these events have not been new to history or unique to Africa. Community resistance must be analysed in context and go beyond simple single-variable determinants. Knowledge and respect of the cultures and beliefs of the afflicted is essential for dealing with threatening disease outbreaks and their potential social violence.

## Introduction

On numerous occasions in the three West African countries most affected by the recent Ebola virus disease (EVD) outbreak – Liberia, Sierra Leone and Guinea – violence erupted, ranging from incidents of rocks thrown at Red Cross vehicles to a massacre leaving eight dead at Macenta. Behind the violence lie deeper causes of distrust – failures of communication, lack of engagement with local communities, adverse publicity, and strained relations between local populations, government authorities, and outside agencies. This distrust and violence provoked concealment of cases, unsafe burial practices, refusal to report contacts, and disruption to clinics, burials, and healthcare. In turn, these factors contributed to the startling spread and persistence of EVD.

Violent resistance to the control of EVD was especially marked in Guinea^1^^,^ 2, but episodes flared in Sierra Leone and Liberia. To list a few, first for Guinea: at Macenta on 5 April 2014, urban youth attacked the town’s first EVD clinic constructed a week earlier, and threatened fifty or more of the centre’s personnel. The protesters claimed EVD did not exist or was spread by outsiders^2^. At Nzérékoré on 29 August, spraying disinfectants through its busy market sparked attacks on the EVD hospital and health workers, leaving twenty-two wounded^2^. Sometime before 27 July, villagers at Wabengou blockaded roads and attacked officials ‘rushing [against] its cars, banging on the vehicles and brandishing machetes’, according to Doctors Without Borders.^3^ Then on 16 September, at Womey, West Africa experienced its worst EVD atrocity when eight members of a high-level delegation of doctors, politicians, and journalists were killed and their bodies dumped in a latrine.^4^ The general level of violence was, however, more pervasive than reported in the international press. In Guinea alone, reported attacks against Red Cross volunteers averaged ten per month in the last six months of 2014^2^.

Although no violence comparable to Womey’s happened in Liberia or Sierra Leone, EVD sparked in these countries open and collective attacks. At Matainkay, east of Freetown, Sierra Leone, on 20 September 2014, villagers assailed health workers while they buried EVD victims, and in December, the Red Cross reported further attacks on their burial teams with damages to their vehicles.^5^ In Liberia at Westpoint, a poor township in Monrovia, an angry mob overran a health care facility, brought out all patients isolated there and looted the clinic.^6^

Such reports may give an impression of a violent, irrational people, resisting health care workers and government officials, whose only wish had been to aid an afflicted people. But how do we explain this resistance, and how have West Africans understood their actions? Were such reactions to epidemic disease new, specific to EVD, or to Africa? Are there historical precedents that can aid our understanding of the ways certain epidemic diseases have provoked conflicts and violence during epidemics?

## Ebola Virus Disease and Cholera

Suspicions and violence against health workers during epidemics have not been unique to Africa, EVD, or the recent past. Instead, the historical record clearly shows that while some diseases, even with rapid contagion or high levels of mortality, brought societies together as with Yellow Fever in nineteenth-century America^7^ and the Great Influenza of 1918-19 globally^8^^,^ 9, others have possessed potent social toxins. One to have riddled European history with violence was cholera and not only during its first pan-European tour of the 1830s. In places such as Italy and Russia, this violence continued into the twentieth century and the number of communities that revolted even expanded over time^8^.

Certain comments and protest slogans, such as ‘Ebola is a lie!’^4^ and ‘Here, if the people come in [to the hospital], they don’t leave alive’^3^ recall nineteenth-and-early-twentieth-century cries during cholera riots in Europe. First, even though Ebola is a virus and cholera’s agent, a bacterium, similarities between the two are striking. Their major symptoms include diarrhoea and vomiting, which cause rapid dehydration; both have manifested extraordinary high case fatality rates of 50 per cent and often more (at least in Africa for the former, and before the twentieth century for the latter)^8^^,^ 10; and the socio-psychological reactions of both have led to collective violence as witnessed with EVD in Guinea, Liberia, and Sierra Leone. Levels of violence and destruction with cholera in Europe greatly exceeded those recently seen with EVD in Africa: for the former, crowds numbering as high as 10,000 rioted and could destroy entire cities as at Hughesofka (present-day Donetsk) and as late as 1892 and with those killed by rioters and subsequent state repression numbering in the hundred.^11^

In the first pan-European cholera wave of the 1830s, riots spread across Eastern and Western Europe from Sicily to Baltic regions, from central Russia to Ireland and across the Atlantic to cities in South and North America. Moreover, no epidemics have provoked such violent antipathy to the medical profession as cholera (the Black Death of 1348 included), with the murder of physicians, surgeons, pharmacists, and nurses and the ritualistic destruction of hospitals and medical equipment^8^^,^ 12 ^,^ 13 ^,^ 14 ^,^ 15 ^,^ 16 ^,^ 17 ^,^ 18.

Across strikingly different cultures, economies, and regimes, the content and character of cholera conspiracies, mistrust and the targets of rioters’ wrath were uncannily similar. Without any obvious communication among rioters from New York City to villages in Asiatic Russia similar stories of elites in conjunction with medical professionals masterminding culls of the poor with wilful poisoning repeated themselves and triggered hundreds of riots (see references above).

Few have studied the persistence of cholera’s core myths and class hatred. As late as 1911, during Italy’s last major cholera wave, over twenty riots erupted, comprised of impoverished workers, fruit and vegetable cultivators, fishermen, women and children sparking crowds up to three thousand to burn down cholera hospitals, level town halls and other government buildings, and murder doctors, pharmacists, mayors, army generals, and carabinieri. As with recent EVD, the rioters pictured themselves as defending their relatives and neighbours against imagined state-led plots executed by doctors to poison their communities. The riots concentrated in mainland Southern Italy but also occurred in northern towns as at Segni, north of Rome in October, 1911, when 3,000 attacked doctors, cholera hospitals and municipal buildings^8^^,^ 19.

By the late nineteenth century Cholera protest had also evolved in other directions with quarantine regulations provoking demonstrations and violence. For instance, at the seaside town Civitavecchia, north of Rome, a crowd of 1,800 of ‘tourists and visitors of all classes’ rioted in 1884 against these violations to their freedom. They besieged railway stations and took ‘a freight train by storm’.^19^ More often, similar to ‘the common sense’ reactions in villages and city districts in Guinea in 2014^2^, protests concerning quarantine cut in the opposite direction: populations rioted because the state had refused to erect barriers to protect communities. Vigilantes led brutal attacks against those escaping infected places as at Monreale (near Palermo) in 1887. Armed with rifles, the monrealesi attacked those fleeing cholera-ridden Palermo, forced the evacuees to camp in fields and stabbed a boy, ‘driven by hunger’, to death.^20^

As with the violence during recent Ebola riots, the reactions and motives of cholera protesters cannot always be described as ‘irrational’. As early as 1867, ‘mobs’ at Naples attacked government offices because of the national government’s incompetence and irresponsibility in improving the city’s sanitary conditions and infrastructure after years of false promises^21^. Yet alongside new protests, the old ones have persisted8, even during the present seventh cholera wave beginning in the 1960s. In 1992, for instance, the Venezuelan government blamed cholera’s spread on the poor’s dirty, uncivilized habits, especially their diet of crabs, while the poor accused the government and multinationals of poisoning their food (especially their crabs) to kill them off^22^. In Brazil similar blame from elites completely neglected the socio-economic roots of cholera and led to the resistance of impoverished residents against the government’s preventive measures^23^.

However, in other countries – the United States, France, and Britain – cholera riots virtually disappeared after 1832. Clearly, these societies were more successful in easing distrust and class tension brought on by the horrors of cholera. With passage of the Anatomy Act in July 1832, for instance, the British state aimed to reduce mounting fears held by the poor that cholera was a state invention to kill them off and to supply cadavers for the new anatomy schools^17^. With subsequent cholera waves in the British Isles, only a handful of small riots numbering in the teens, and not the thousands appeared. Yet these differences across time and place have yet to be studied^8^.

One hypothesis explaining the longevity of cholera riots in Eastern Europe points to the state’s continued ruthless intervention and its draconian quarantine regulations^24^. But, in Spain, Portugal, and Italy from the 1860s to the end of the century, the reason for cholera riots was often the opposite: state reluctance to impose quarantines sparked crowds to revolt.

## Understanding resistance in Liberia

In Liberia various forms of community resistance were reported against measures of surveillance, quarantine, isolation, and treatment, such as denying the disease existed, spreading rumours that EVD was transmitted by intentionally poisoning wells or food, that NGOs or the government had spread it to make money, that NGOs used body parts of EVD victims for profit, that whites had introduced EVD to stop the hunting of apes in forests. As a result, on several occasions Liberians refused to obey quarantine regulations, to report or isolate the afflicted, performed secret and unsafe burials, and, as seen above, attacked health care workers and health facilities.

Community resistance to EVD regulations must be analysed in context. Towards the end of 2014, after the epidemic peaked, the government introduced new policies for contact tracing and active case finding. Hundreds were forced to remain quarantined in their homes, often without adequate food and water. Many were forced to violate the quarantine to buy basic provisions in the markets in order to survive.^25^ Living conditions among those quarantined were often overcrowded, with lack of basic sanitation and no running water. Add to that the constant fear that a beloved one or indeed oneself might be developing symptoms of a disease that only few survived.

Other inconveniences and insensitivity on the part of the government and health agencies made matters worse for afflicted communities. For instance, at the peak of the epidemic, when ambulances picked up those suspected of being infected and carried them to Ebola Treatment Units (ETUs), health authorities rarely informed the families where their relatives had been taken or if they had tested positive. Authorities failed even to inform families if their loved ones had survived or died, causing local populations to believe that patients simply ‘disappear in the ETUs’. As a result, these centres were seen as death camps from which no one returned alive. Rumours spread that the victims had not died of Ebola but had been murdered in the ETUs to harvest their blood and organs. Such tales were similar to those underlying cholera riots, especially in 1832 in the British Isles, when hospitals were seen as abattoirs producing body parts and cadavers for medical instruction. At Monrovia, a large U.S. army ship, anchored in its harbour, was perceived to collect body parts of the Ebola dead to benefit Americans. Stories circulated of patients being neglected in ETUs or starved to death^26^, and government and health agencies unwittingly spread negative publicity about EVD and its spread, declaring it was a deadly disease without cure. Such messages fanned fear, contributing, no doubt, to the resistance sketched above.

Increasingly, relatives who died at home were not reported to authorities and buried secretly. These burials were often unsafe and a key vector to new infections. Secret burials mounted in number, because of fear of being quarantined or stigmatized. Furthermore, the enforcement of ‘safe burials’ violated communities’ religious beliefs and customs^27^. By August 2014, fears worsened when the government ordered all corpses cremated: serious resistance now spread to many communities. Police and military forces were deployed along the road leading to the crematory to protect the “Ebola burners”^28^. However, the government was accused of being inconsistent in how they performed burial practices. Financial ability or social connections would determine who was cremated and who was buried^27^^,^ 29. The order remained in force until January 2015, when finally a burial ground was created near the Capital, Monrovia.

Such occurrences find historical parallels with cholera, when municipalities also prohibited traditional funerals, burials in churchyards, and especially the Irish wake. Enforcement of these regulations (as in West Africa) led to concealing cases and provoked large-scale riots among immigrants in Bristol, London, Liverpool, Glasgow, Edinburgh, New York City, and other towns during the first European cholera wave. Similar measures had similar consequences in Asiatic Russia, Eastern Europe, the Iberian peninsula, and Italy; yet here governments’ insensitivity persisted longer. Space allows only a brief look at an Italian case from its first cholera wave in 1836-7 to its last major one in 1910-11. Throughout, authorities continued to prohibit non-elites (il popolo) from performing their traditional burial rites, visiting afflicted friends and relations, and viewing the cadavers before burial. Such class-based impositions sparked fears that doctors backed by the state had poisoned the cholera afflicted or had buried them alive, while Italian elites continued to bury their love ones stricken by cholera in traditional ecclesiastical grounds. Seeing their relatives unceremoniously ‘thrown into ditches’ of newly-created cholera grounds outside towns and villages, the popolo in Sicily rioted in numerous places in 1837^30^. The same ensued at the Pugliese town of Ostuni in 1837 with its popolo shouting they would not tolerate it.^31^ But Italian authorities appear not to have learnt the lessons. During Italy’s last major cholera wave in 1910-11, the state continued to impose the same burial restrictions, provoking fears of poisoning and live burials. But now Ostuni’s collective violence exploded beyond its previous experiences. In mid-November 1910, 3,000 (in a town numbering 18,500) wrecked the cholera hospital, ‘liberated the patients’, paraded them home, burnt down the town hall and offices of the health department, took possession of the town square, attacked health workers, stoned carabinieri, and destroyed doctors’ homes^32^^,^ 33.

Back to Liberia: considering certain government measures, the insensitivity in which they were implemented, the lack of support, and comparisons with Europe’s past, it is surprising that more people did not resist openly. Instead, most Liberians cooperated with the response teams. In the beginning of the epidemic with people dying in the streets, not enough space was available in ETUs, and when a 120-bed ETU in Monrovia opened on 21 September 2014 it became full within hours.^34^ Often transport was unavailable, and patients were forced to wait days for ambulances or were carried to ETUs by taxis, bikes, or in wheelbarrows. ETUs were understaffed, and everywhere essential materials were lacking. Matters began improving only after the epidemic had peaked: international support began to arrive, and several new ETUs were built.

## Community engagement****

In virtually all former EVD epidemics in East and Central Africa, violence, community resistance or suspicion was reported, and these experiences resembled those in 2014/15.^35^^,^ 36 ^,^ 37 Anthropologists recommended that in future epidemics communities should be encouraged to be more involved in the medical response so that inhabitants would be seen as allies, not as enemies. In the recent epidemic, the same is demanded: public health measures work only when authorities listen to the affected communities and involve them non-paternalistically, allowing confidence in the health system to develop^26^^,^ 38 The WHO Ebola Interim Assessment Panel in its final report from July 2015 expressed its surprise and dismay on the lack of engaging local communities in the response and that ‘culturally sensitive messages and community engagement were not prioritized’.^39^ While in the WHO’s document ‘Roadmap for Action’^40^ community engagement is merely an addendum, the 2015 WHO Strategic Response Plan^41^ stresses community engagement mainstreaming as one important pillar in the Ebola response with clear outputs and key activities. As a matter of fact, WHO was the first organisation to employ anthropologists in Ebola response teams, recognizing that EVD containment should not only be seen bio-medically but also from a socio-cultural perspective. In the retrospective joint report by Harvard University and the London School of Hygiene and Tropical Diseases^42^ the poor understanding of the importance of community engagement is mentioned as a system weakness but this critique did not lead to any recommendations. Violence, serious social disruptions or workforce- and community safety are not explicitly mentioned in any documents, nor is their historical context.

In conclusion the recent adverse reactions to EVD in Western Africa have not been new to history or unique to Africa. Instead, the distrust and violence seen in Guinea, Liberia, and Sierra Leone in 2014/15 parallel Europe’s long encounters with cholera from 1830 into the twentieth century. This European past combined with recent events in West Africa can provide lessons for policy makers. First, authorities and health workers must recognize that some diseases have greater potential to spark distrust and violence than others, and the former tend to be ones with high fatality rates, when victims enter health facilities but few return alive. Second, responsible public health experts must negotiate carefully funeral and burial practices central to the ritual life of Africans as they have been for Europeans. Regulating these matters mindful of infection prevention and epidemiological factors alone, without engaging the afflicted communities or their leaders, not only risks triggering resistance, distrust, and violence; it threatens fanning the spread of the epidemic beyond control, increasing misery and the numbers dying.

## Competing Interest

The authors have declared that no competing interests exist.

## Disclaimer

The opinions expressed here are those of the authors and do not necessarily reflect the positions of WHO.
